# Gout and risk of chronic kidney disease and nephrolithiasis: meta-analysis of observational studies

**DOI:** 10.1186/s13075-015-0610-9

**Published:** 2015-04-01

**Authors:** Matthew J Roughley, John Belcher, Christian D Mallen, Edward Roddy

**Affiliations:** School of Medicine, Keele University, Keele, Staffordshire ST5 5BG UK; School of Computing and Mathematics, Keele University, Keele, Staffordshire ST5 5BG UK; Arthritis Research UK Primary Care Centre, Keele University, Keele, Staffordshire ST5 5BG UK

## Abstract

**Introduction:**

To determine the prevalence of chronic kidney disease and nephrolithiasis in people with gout, and the association between gout and prevalent or incident chronic kidney disease and nephrolithiasis.

**Methods:**

Systematic review and meta-analysis of epidemiological studies. Data sources; MEDLINE, EMBASE and CINAHL databases, hand-searched reference lists, citation history and contact with authors. Eligibility criteria: cohort, case–control or cross-sectional studies which examined the occurrence of chronic kidney disease or nephrolithiasis amongst adults with gout (with or without a non-gout comparator group) in primary care or general population samples. Prevalence and risk estimate meta-analyses were performed using a random-effects model.

**Results:**

Seventeen studies were included in the meta-analysis (chronic kidney disease n = 7, nephrolithiasis n = 8, both n = 2). Pooled prevalence estimates of chronic kidney disease stage ≥3 and self-reported lifetime nephrolithiasis in people with gout were 24% (95% confidence interval 19% to 28%) and 14% (95% CI 12% to 17%) respectively. Gout was associated with both chronic kidney disease (pooled adjusted odds ratio 2.41, 95% confidence interval 1.86 to 3.11) and self-reported lifetime nephrolithiasis (1.77, 1.43 to 2.19).

**Conclusions:**

Chronic kidney disease and nephrolithiasis are commonly found amongst patients with gout. Gout is independently associated with both chronic kidney disease and nephrolithiasis. Patients with gout should be actively screened for chronic kidney disease and its consequences.

## Introduction

Gout is the most prevalent inflammatory arthritis, affecting 2.4% of adults in the UK [1]. Gout is associated with considerable co-morbidity including hypertension, diabetes mellitus, obesity, metabolic syndrome, and vascular disease [2]. Associations between gout and renal disease and nephrolithiasis have long been recognised, yet early studies undertaken in specialist secondary care populations are likely to be unrepresentative of most patients with gout who are managed exclusively in primary care settings [3-6]. To the best of our knowledge, no previous systematic reviews examining this association have been performed.

Chronic kidney disease (CKD) stages 3 to 5 (glomerular filtration rate <60 ml/minute/1.73 m^2^) affects 8.5% of the UK population, is more common in females than males (10.6% vs. 5.8%), and is associated with both all-cause mortality and cardiovascular disease [7,8]. If left untreated, CKD may progress to end-stage renal disease (ESRD) requiring expensive renal replacement therapy (RRT) [9]. CKD is an independent risk factor for the development of gout [10,11] yet there are several reasons to explain why gout may predispose to renal disease, including hyperuricaemia, chronic inflammation, co-morbid hypertension and diabetes mellitus, and use of nonsteroidal anti-inflammatory drugs. The management of gout in patients with renal disease is challenging because nonsteroidal anti-inflammatory drugs and colchicine should be used with caution and lower doses of allopurinol are required [12].

The lifetime prevalence of nephrolithiasis is approximately 8.8% [13]. Kidney stones most commonly present as renal colic but can be complicated by infection, renal tract obstruction requiring surgical intervention, and renal failure [14]. Gout is associated with lower urinary pH theoretically predisposing to the formation of both uric acid and other stones [5,15]. Diabetes mellitus, obesity, and hypertension are also known to be independent risk factors for nephrolithiasis [16-18].

The objectives of this systematic review and meta-analysis of epidemiological studies were to quantify the prevalence of CKD and nephrolithiasis in gout, and to determine whether gout is associated with prevalent or incident CKD and nephrolithiasis in the general population.

## Methods

### Data sources and searches

A systematic literature search was undertaken by two co-investigators (MJR and ER) in the MEDLINE, EMBASE, and CINAHL databases using the National Health Service’s Healthcare Databases Advanced Search from inception date to November 2013 for epidemiological studies examining the association between gout and either CKD or nephrolithiasis. Search terms pertaining to gout were combined with terms describing the outcome (CKD or nephrolithiasis) and observational study design using the Boolean operator “AND”. No unpublished data sources were searched. The references and citation history of all relevant studies were checked for additional data sources.

### Eligibility criteria

Eligible studies were required to include adults with gout (with or without a nongout comparator group), describe the occurrence of CKD or nephrolithiasis amongst adults with gout (and nongout comparator group where included), be of an epidemiological design (cross-sectional, cohort, case–control), and have been undertaken in a primary care or general population sample. No geographical or language restrictions were imposed. Conference abstracts were not included.

### Study selection

MJR and ER independently screened the title and abstract of all identified studies. Studies which could not be excluded on title/abstract review were retained for full-text review undertaken by the same reviewers. At both the title/abstract and full-text review stages, disagreement over study eligibility was resolved by consensus discussion between the reviewers. A third reviewer (CDM) was available where disagreement could not be resolved by consensus.

### Data extraction and quality assessment

The following data were extracted independently by the same two reviewers from studies meeting the inclusion criteria: date and location of study, study population and size, study type, demographic characteristics (age and gender), method of ascertainment of gout and CKD/nephrolithiasis, prevalence and incidence of CKD/nephrolithiasis in gout and comparator populations, unadjusted and adjusted risk estimates (odds ratio, relative risk, hazards ratio) and corresponding 95% confidence interval (CI) or standard error, and details of covariates included in multivariate models. Studies utilising any method of gout ascertainment (for example, crystal identification, classification criteria, clinical diagnosis) were included. Staging of CKD was defined according to the National Kidney Foundation classification (stage 1, glomerular filtration rate ≥90 ml/minute/1.73 m^2^; stage 2, glomerular filtration rate 60 to 89 ml/minute/1.73 m^2^; stage 3, glomerular filtration rate 30 to 59 ml/minute/1.73 m^2^; stage 4, glomerular filtration rate 15 to 29 ml/minute/1.73 m^2^; stage 5 (ESRD), glomerular filtration rate <15 ml/minute/1.73 m^2^) [19]. Assessment of methodological quality was performed independently by the same two reviewers using the Newcastle–Ottawa quality appraisal tool with additional items regarding the method of diagnosis of gout, CKD, and nephrolithiasis [20]. Disagreement over data extraction and assessment of methodological quality was resolved by consensus discussion between the reviewers. Authors were contacted to request additional information and data where necessary.

### Data analysis

Estimates of point/lifetime prevalence of CKD/nephrolithiasis in adults with gout and, where possible, risk estimates of the association between gout and CKD/nephrolithiasis were calculated. These estimates were derived from summary 2 × 2 tables, together with regression estimates arising from adjusted analyses using logistic and Cox regression.

To normalize the distribution, prevalence rates were transformed by means of the logit event rate:

$$ \mathrm{L}\mathrm{p} = \mathrm{L}\mathrm{n}\left(\mathrm{p}\ /\ \left(1\ \hbox{-}\ \mathrm{p}\right)\right) $$

with p being the prevalence rate, Ln the natural logarithm, and Lp the logit event rate. The sampling variance of each logit event rate, V(Lp), was calculated by means of:

$$ \mathrm{V}\left(\mathrm{L}\mathrm{p}\right) = 1\ /\ \left(\mathrm{n}\mathrm{p}\right) + 1\ /\ \left(\mathrm{n}\left(1\ \hbox{-}\ \mathrm{p}\right)\right) $$

with n being the sample size. Once the statistical analyses were carried out, the results were back transformed to prevalence rates to facilitate their interpretation.

Separate analyses were undertaken for CKD and nephrolithiasis. Where sufficient data were present in individual studies but risk estimates were not reported, these were calculated using the available data. Pooled estimates were calculated using a random effects model. The 95% CIs and forest plots were produced for all pooled estimates.

Heterogeneity was assessed visually with forest plots, and quantified numerically using the *I*^2^ index [21] and Cochran’s *Q* test. The *I*^2^ test describes the percentage of variation across studies due to heterogeneity rather than chance. Meta-analyses were performed using the *metan* command within STATA 12.1 (StataCorp, College Station, TX, USA).

## Results

### Study selection

**Figure 1:**
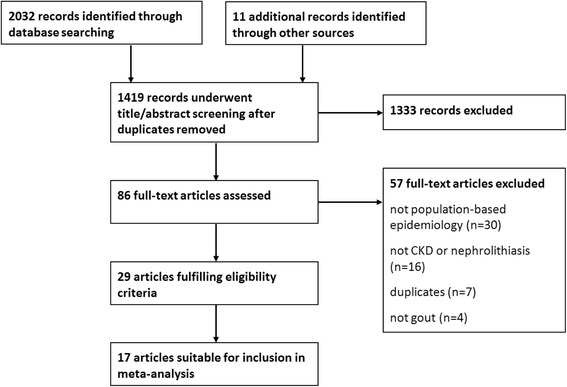
Selection of studies included in the review. CKD, chronic kidney disease.

The search identified 2,032 potentially relevant articles: after removal of duplicates, 1,419 remained (Figure 1). Following title/abstract review, 1,333 articles were excluded. Of the 86 articles remaining, 57 were excluded after full-text review and 29 met the eligibility criteria. Seventeen studies contained data suitable for pooling. Reasons for exclusion are shown in Figure 1.

### Study characteristics

Twelve studies examined CKD [22-33], 15 studies examined nephrolithiasis [13,34-47], and two studies examined both CKD and nephrolithiasis [48,49] (Table 1). Nineteen cross-sectional studies [13,22,25,27,29,30,32,34-38,40-42,46-49], one case–control study [26], and nine cohort studies [23,24,28,31,33,39,43-45] were identified. Six of the nine cohort studies included relevant outcome data only at baseline and so were treated as cross-sectional studies [24,31,33,43-45].

**Table 1:** **Characteristics of included studies**

**First author**	**Study period**	**Location**	**Source of data/study population**	**Study type**	**Total sample**	**Mean age (SD)** ^**a**^	**% male**	**Gout ascertainment**	**Outcomes reported (method of ascertainment)**
Chen [22]	2006	China	Residents of Changning district of Shanghai	CS	2,554	58.4 (SE 15.3)	36	Self-report	Adjusted ORs for CKD any stage and CKD stage ≥3 (biochemical measurement)
Hsu [23]	1964 to 2000	USA	Kaiser Permanente Northern California linked to US Renal Data System	Cohort	177,570	ESRD 42.3 (10.3)	ESRD 59	Self-reported physician diagnosis	Univariate and multivariate HRs for ESRD requiring RRT (record linkage)
						No ESRD 40.7 (14.0)	No ESRD 46		
Kuo [24]	2000 to 2006	Taiwan	Chang Gung Memorial Hospital health screening programme	CS data from cohort^b^	61,527	Males 49.1 (11.0)	56	Self-reported or ICD code or crystal identification	Prevalence of CKD stage ≥3 (biochemical measurement)
						Females 50.8 (10.8)			
Fuldeore [25]	2002 to 2005	USA	Managed Healthcare Database	CS	3,929	CKD	CKD	ICD code on two occasions or ICD code plus gout medication	Prevalence of CKD stage 2, 3 and 4 (biochemical measurement)
						54.3 (NR)	86		
						No CKD	No CKD		
						47.8 (9.0)	89		
Johnson [26]	1997 to 2004	USA	Kaiser Permanente North West	CC	5,335	64.4 (NR)	46	ICD code or SUA ≥7 mg/dl	Unadjusted and adjusted ORs for ESRD requiring RRT (medical record review)
Keenan [27]	2007 to 2008	USA	New York Veterans Affairs database	CS	575	71.8 (11.6)	99	ICD code	Prevalence of CKD stage ≥3 (biochemical measurement or ICD code)
Yu [28]	2000 to 2008	Taiwan	Taiwan National Health Insurance Database	Cohort	656,108	41.1 (15.6)	73	ICD code on two occasions or ICD code plus gout medication	Multivariate HR for ESRD requiring RRT (record linkage)
O’Sullivan [34]	1964	USA	Population of Sudbury, Massachusetts	CS	4,626	NR	NR	New York/Rome criteria	Prevalence of nephrolithiasis (self-report, not defined)
Schaffalitzky De Muckadell [35]	1973	Denmark	Male office workers aged 40 to 59 years in Copenhagen	CS	312	NR	100	Self-reported physician diagnosis	Prevalence of nephrolithiasis (self-report, not defined)
Currie [36]	1969 to 1975	UK	General practice records	CS	604	52.3 (NR)	77	GP diagnosis	Prevalence of nephrolithiasis (GP diagnosis)
Currie [37]	1976	UK	General practice records	CS	64,454	NR	NR	GP diagnosis	Incidence of nephrolithiasis (GP diagnosis)
Kramer [38]	1988 to 1994	USA	National Health and Nutrition Examination Survey (NHANES) III	CS	17,030	Stones 53.7 (0.7)	48	Self-reported physician diagnosis	Prevalence and adjusted ORs for nephrolithiasis (self-reported lifetime prevalence)
						No stones 44.2 (0.4)			
Kramer [39]	1986 to 1998	USA	Health Professionals Follow-up Study	Cohort	51,529	Gout 59.9 (9.1)	100	Self-reported physician diagnosis, ARA criteria	Prevalence and adjusted RRs for nephrolithiasis (self-reported lifetime prevalence)
						No gout 54.4 (9.8)			
Mikuls [40]	1990 to 1999	UK	General Practice Research Database (GPRD)	CS	207,350	Gout 60.5 (15.4)	46	GP diagnosis	Prevalence, unadjusted and adjusted ORs for nephrolithiasis (medical records)
						OA controls 66.8 (13.4)			
Harrold [41]	1999 to 2003	USA	Health Maintenance Organisations (HMO) Research Network Centre for Education and Research on Therapeutics (CERT)	CS	6,133	Males 58 (14)	81	ICD code on two occasions	Prevalence of nephrolithiasis (ICD code)
						Females 70 (12)			
Padang [42]	Not stated	Indonesia	Community Health Centres in Northern Sulawesi	CS	380	NR	NR	≥3 attacks of gout/year and/or tophi plus ARA criteria	Prevalence of nephrolithiasis (self-report, not defined)
Sarawate [43]	1999 to 2004	USA	Managed Care Database	CS data from cohort^b^	5,942	57.4 (14.1)	76	ICD code on two occasions or ICD code plus gout medication	Prevalence of nephrolithiasis (ICD code)
Solomon [44]	Not stated	USA	US Medicare system and Pharmacy Assistance Contract for the Elderly (PACE)	CS data from cohort^b^	9,823	Male 78 (7)	16	Use of ULT	Prevalence of nephrolithiasis (medical records)
						Female 80 (7)			
Harrold [45]	2000 to 2006	USA	Health Maintenance Organisations (HMO) Research Network Centre for Education and Research on Therapeutics (CERT)	CS data from cohort^b^	4,166	62 (14)	75	ICD code plus use of ULT	Prevalence of nephrolithiasis (ICD code)
Roddy [49]	2005	UK	Registered population of two general practices in Nottingham	CS	3,082	Gout 63.8 (10.6)	Gout 81	Self-reported gout, validated by rheumatologist	Prevalence of CKD stage ≥3, ≥4 (biochemical measurement) and nephrolithiasis (self-reported lifetime prevalence)
						No gout 57.0 (14.4)	No gout 41		
Zhu [48]	2007 to 2008	USA	National Health and Nutrition Examination Survey (NHANES)	CS	5,707	47 (NR)	48	Self-reported physician diagnosis	Prevalence and adjusted OR for CKD stage ≥2, ≥3^c^ (biochemical measurement) and nephrolithiasis (self-reported lifetime prevalence)
Liote [29]	2008 to 2009	France	General Practitioner data from GOSPEL survey	CS	810	62.7 (11.3)	87.2	Physician diagnosis	Prevalence of CKD stage ≥3, ≥4 (biochemical measurement)
Lin [30]	2007	Taiwan	Community-based survey, sampling via Household Register Database	CS	3,352	47.5 (17.4)	48.6	Self-reported physician diagnosis of gout or hyperuricaemia	Prevalence of CKD stage ≥3 (biochemical measurement)
Kuo [31]	2000 to 2008	Taiwan	Longitudinal Health Insurance Database	CS data from cohort^b^	704,503	42.73 (16.6)	Gout 70.3, no gout 50.4	ICD code	Prevalence and unadjusted OR for ESRD requiring RRT (record linkage)
Krishnan [32]	2009 to 2010	USA	National Health and Nutrition Examination Survey	CS	5,589	44 (21)	49	Self-reported physician diagnosis	Prevalence of CKD stage 2, 3, ≥4 (biochemical measurement)
Kok [33]	2003 to 2007	Taiwan	Taiwan National Health Insurance Database	CS data from cohort^b^	3,858,840	NR	48.3	ICD code on three occasions	Prevalence of CKD, stages not specified (ICD code)
Trifiro [46]	2005 to 2009	Italy	Health Search/Cegedim Strategic Data Longitudinal Patient Database	CS	12,276	NR	74	ICD code, related keywords for free text search	Prevalence and unadjusted OR for nephrolithiasis (medical records)
Scales [13]	2007 to 2010	USA	National Health and Nutrition Examination Survey	CS	12,110	NR	NR	NR	Adjusted OR for nephrolithiasis (self-reported lifetime prevalence)
Ando [47]	1995 to 2001	Japan	Men undergoing medical examination at Gifu Prefectural Center for Health Check and Health Promotion	CS	13,418	Controls 48.5 (8.8), past stones 49.7 (8.4)	100	Receiving medical treatment for gout or SUA ≥7.0 mg/dl	Prevalence nephrolithiasis (self-reported lifetime prevalence)

ARA, American Rheumatism Association; CC, case–control study; CKD, chronic kidney disease; CS, cross-sectional; ESRD, end-stage renal disease; GP, general practitioner; HR, hazard ratio; ICD, International Classification of Disease; NR, not reported; OA, osteoarthritis; OR, odds ratio; RR, relative risk; RRT, renal replacement therapy; SD, standard deviation; SE, standard error; SUA, serum uric acid level; ULT, urate-lowering therapy. ^a^Assumed to be SD unless otherwise stated. ^b^Cohort study reporting only cross-sectional data relevant to this review. ^c^CKD stages clarified with authors; CSD, Cegedim Strategic Data.

Fourteen studies were performed in the USA [13,23,25-27,32,34,38,39,41,43-45,48], four in the UK [36,37,40,49], five in Taiwan [24,28,30,31,33], and one study each in China [22], Denmark [35], France [29], Indonesia [42], Italy [46], and Japan [47]. One study restricted participation to people aged over 65 years [44] and three studies included only males [35,39,47]. Fourteen studies were undertaken in large healthcare databases [23-28,31,33,40,41,43-46] whereas 15 studies used empirically collected data [13,22,29,30,32,34-39,42,47-49].

### Assessment of methodological quality

Only three of 29 studies based the diagnosis of gout on validated clinical classification criteria: two studies [39,42] used the American Rheumatism Association criteria [50], and one study [34] used the New York and Rome criteria [51,52]. No studies based the diagnosis of gout on crystal identification. Three studies reported combined outcomes for gout and hyperuricaemia [26,30,47]. Nine studies used biochemical measures to define CKD [22,24,25,27,29,30,32,48,49], four studies examined ESRD requiring RRT [23,26,28,31], whereas nine of 17 studies of nephrolithiasis defined outcome by self-report [13,34,35,38,39,42,47-49], six studies used medical record linkage [40,41,43-46], and two studies used a general practitioner completed questionnaire [36,37].

Participants were considered not representative of the typical community gout patient in eight studies due to including only patients prescribed urate-lowering therapy [25,44,45], only males [35,39,47], or only people with chronic gout [42] and one study being undertaken in a Veterans database which resulted in a predominantly male (99%) older population (mean age 72 years) [27]. All studies featuring a nongout comparator group had drawn this from the same community as the exposed cohort. Response and data usage rates were frequently not reported [13,26,38,41-45,48], Two surveys reported response rates under 60% [30,49] and two database studies based in a managed care setting used less than 70% of records available to them [24,31].

Of the three cohort studies, none reported rates of attrition and one did not state whether the outcome (ESRD) was absent at the start of the study [23]. Length of follow-up was deemed adequate to determine outcome in all three studies.

### Selection for meta-analysis

Seventeen out of 29 studies were included in the meta-analysis [13,22-24,28-30,32,34,35,38,39,42,46-49]. Two studies examining CKD were deemed unsuitable for pooling due to use of diagnostic codes [27,33] rather than biochemical testing to ascertain CKD that has been shown to significantly underestimate prevalence [53,54]. One study demonstrated significant incompleteness of biochemical data and was not pooled [25]. Ten out of 17 studies examining self-reported nephrolithiasis were pooled [13,34,35,38,39,42,46-49], and six studies were deemed too methodologically different to pool due to using a general practitioner completed questionnaire [36] or diagnostic codes and/or a limited time period for ascertaining nephrolithiasis [40,41,43-45]. Three studies reported outcomes that were not reported by other studies, thus precluding meta-analysis [26,31,37].

### Prevalence and risk of chronic kidney disease in gout

**Figure 2:**
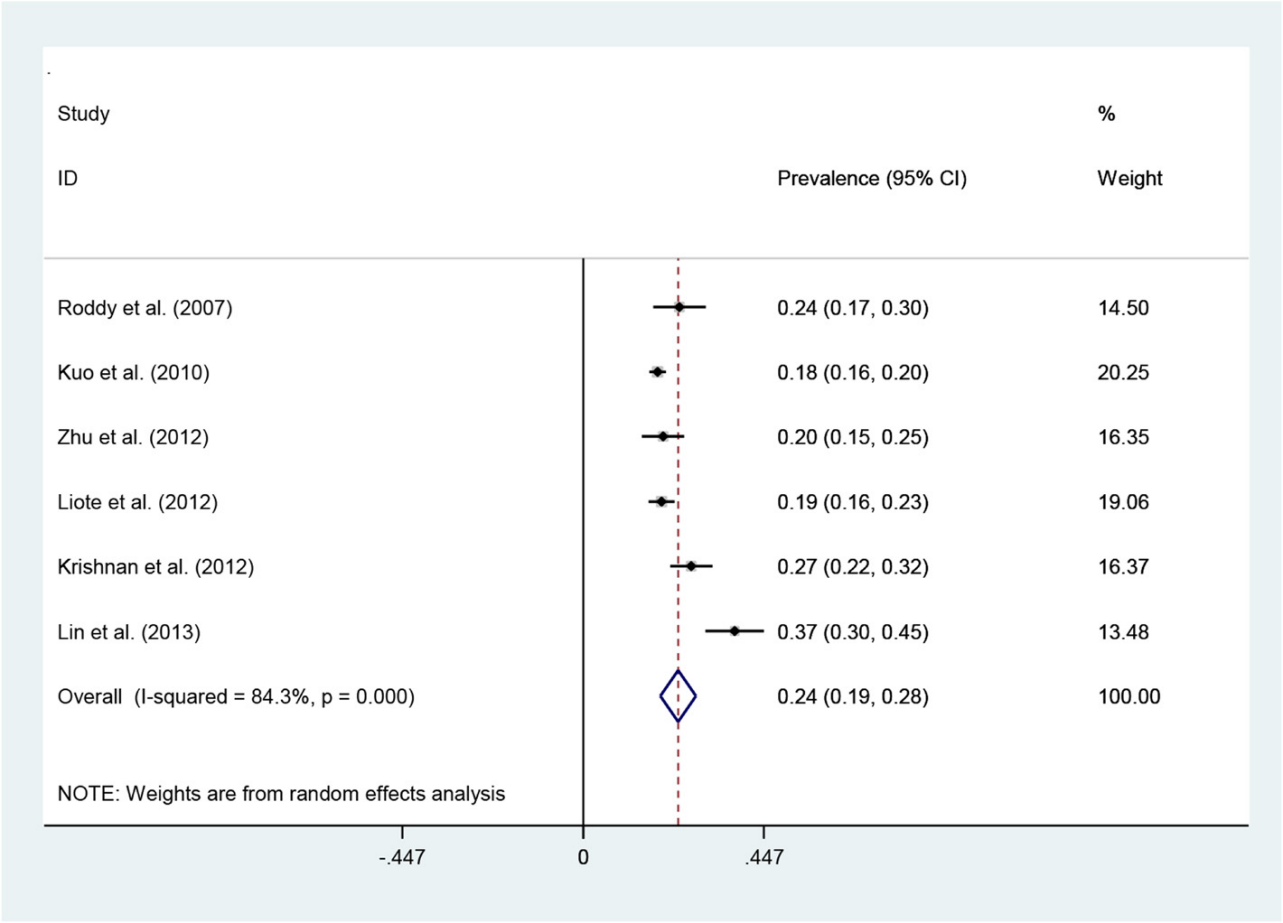
Forest plot of individual and pooled (random effects) prevalences of chronic kidney disease stage ≥3 in gout. CI, confidence interval.

Six studies provided suitable data to allow the pooled prevalence of CKD stage ≥3 in people with gout to be calculated [24,29,30,32,48,49]. Further data [29,30] and outcome clarification [48] was requested from the authors of three of these studies, enabling their inclusion. No reply was received from the authors of a further paper from whom the prevalence of CKD in those with and without gout was requested. The pooled prevalence estimate of CKD stage ≥3 in people with gout was 24% (95% CI 19%, 28%) (Figure 2). Statistically significant heterogeneity was identified (*I*^2^ = 84.3%, *P* <0.001). Data from three studies [29,32,49] were pooled providing a prevalence estimate of CKD stage ≥4 in gout of 2% (95% CI 0%, 4%) (*I*^2^ = 82.5%, *P* = 0.003).

**Figure 3:**
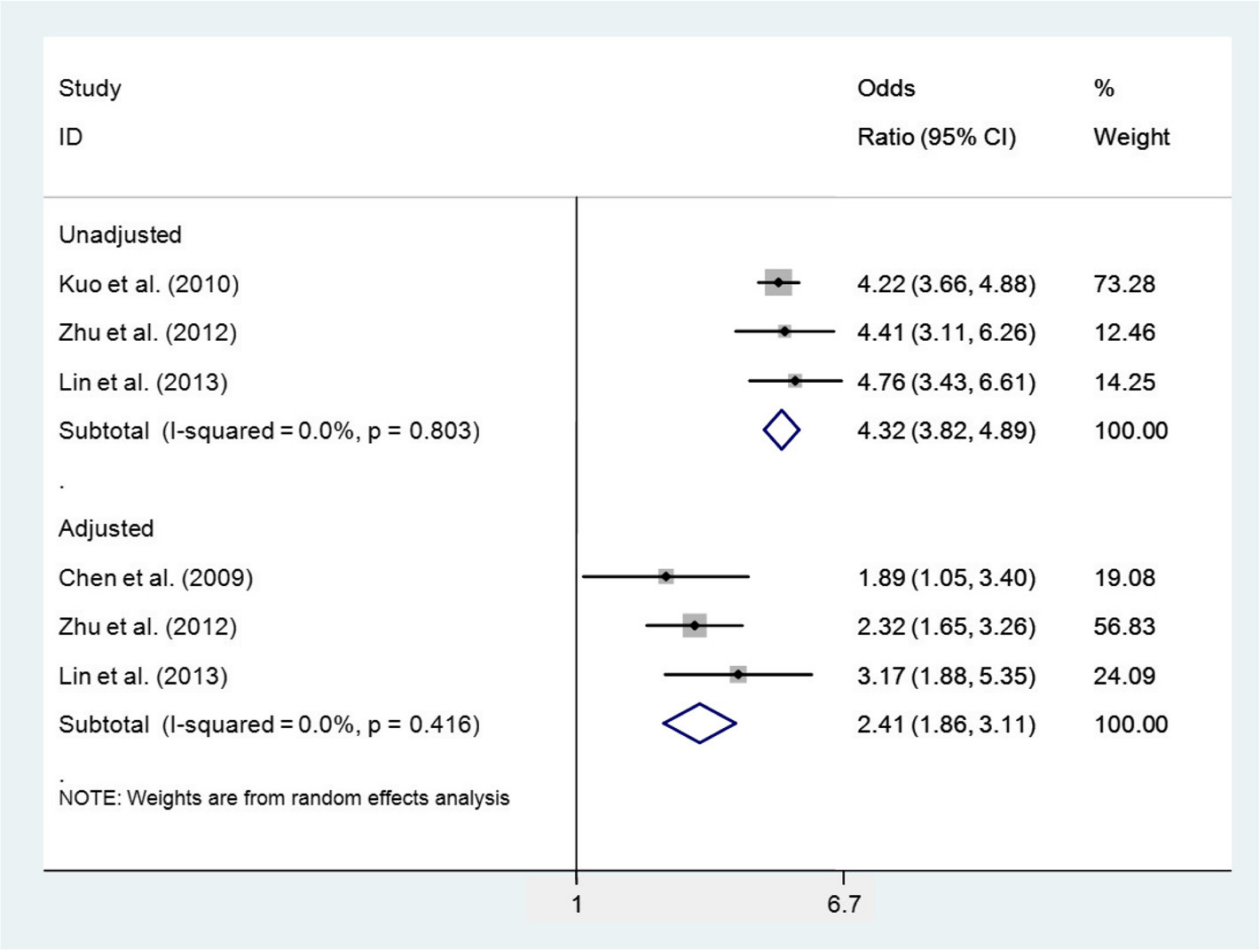
Forest plot of individual and pooled (random effects) unadjusted and adjusted odds ratios between gout and chronic kidney disease stage ≥3. CI, confidence interval.

Comparing patients with gout to those without gout, the pooled unadjusted odds ratio (OR) between gout and CKD stage ≥3 was 4.32 (95% CI 3.82, 4.89) (Figure 3) based on the findings of three studies [24,30,48]. Pooling an age-adjusted and gender-adjusted OR and two multivariate ORs (adjustment included age, gender, obesity, hypertension, and diabetes mellitus) between gout and CKD stage ≥3 resulted in an adjusted OR of 2.41 (95% CI 1.86, 3.11) (Figure 3) [22,30,48]. There was no significant heterogeneity identified for either pooled unadjusted or adjusted ORs (unadjusted *I*^2^ = 0.0%, *P* = 0.803; adjusted *I*^2^ = 0.0%, *P* = 0.416).

One study reported prevalence of ESRD requiring RRT in gout (0.6%) and provided an unadjusted OR for ESRD in gout versus no gout (OR 2.65; 95% CI 2.25, 3.12) [31]. One retrospective matched case–control study compared the likelihood of a prior combined exposure of gout or hyperuricaemia between cases with ESRD requiring RRT and controls without ESRD matched for age, gender, and year of RRT (adjusted OR 2.51; 95% CI 1.78, 3.54) [26].

Following contact with the authors of one study to provide an unadjusted hazard ratio (HR) (95% CI) between gout and ESRD [28], there were two prospective cohort studies [23,28] that provided suitable data to allow a pooled unadjusted HR between gout and incident ESRD to be calculated (HR 2.69; 95% CI 1.00, 4.39). Mean follow-up periods were 24.5 years in one study [23] and 8.0 years overall in the other [28]. Significant heterogeneity was identified (*I*^2^ 93.1%, *P* <0.001). We contacted the authors of one of these studies to request an adjusted HR (95% CI) between gout and incident ESRD [23]. The authors replied but were unable to provide this information. Only one study provided a multivariate adjusted HR between gout and incident ESRD (HR 1.57; 95% CI 1.38 to 1.79; adjusted for age, sex, diabetes and hypertension) [28].

### Prevalence and risk of nephrolithiasis in gout

**Figure 4:**
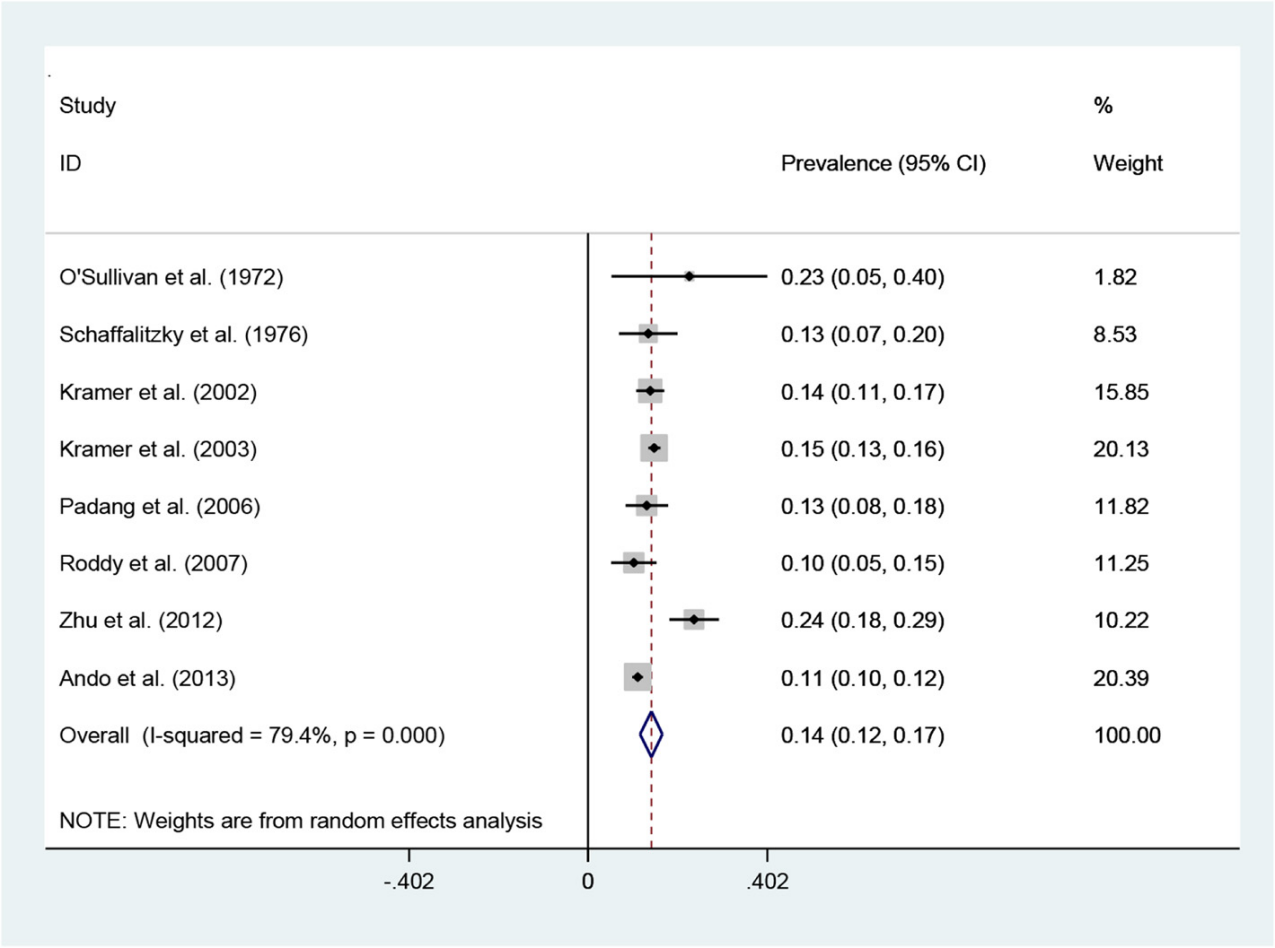
Forest plot of individual and pooled (random effects) prevalences of nephrolithiasis in gout. CI, confidence interval.

We were able to use additional unpublished data from one of our previous studies [49] concerning the prevalence of self-reported lifetime nephrolithiasis in people with gout and ORs (95% CI) between gout and self-reported lifetime nephrolithiasis, unadjusted and then adjusted, firstly, for age and gender and, secondly, for age, gender, diabetes, hypertension, and diuretic use. The pooled estimate of the prevalence of nephrolithiasis in people with gout was 14% (95% CI 12%, 17%) (Figure 4) based on the findings of eight studies [34,35,38,39,42,47-49]. Significant statistical heterogeneity was identified (*I*^2^ = 79.4%, *P* <0.001).

**Figure 5:**
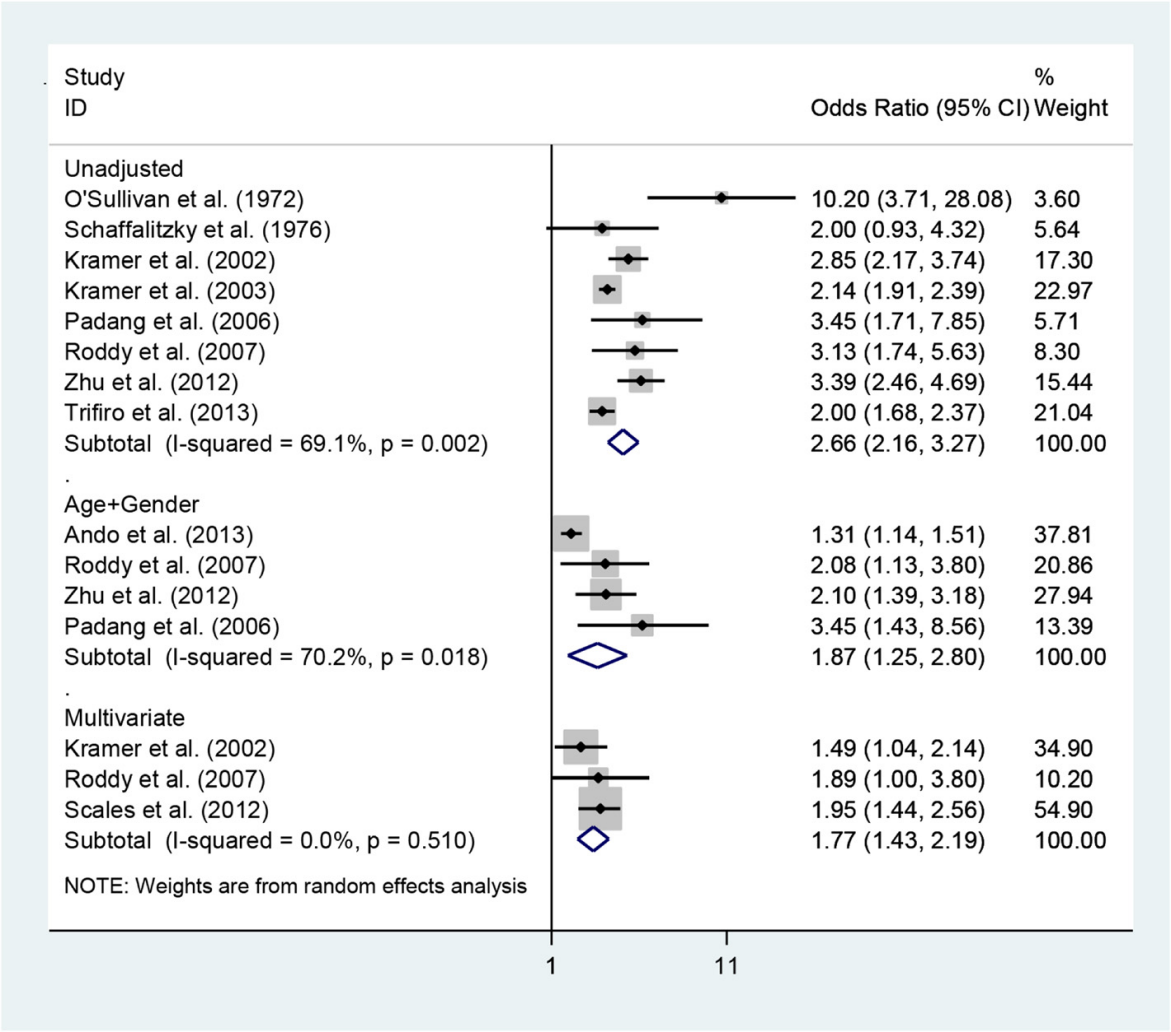
Forest plot of individual and pooled (random effects) unadjusted and adjusted odds ratios between gout and nephrolithiasis. CI, confidence interval.

Eight studies [34,35,38,39,42,46,48,49] provided suitable data to calculate a pooled unadjusted OR between gout and self-reported prevalence of nephrolithiasis (OR 2.66; 95% CI 2.16, 3.27) (Figure 5). The pooled age-adjusted and gender-adjusted OR was 1.87 (95% CI 1.25, 2.80), based on the findings of four studies [42,47-49]. Three studies provided suitable data to permit a pooled multivariate adjusted OR to be calculated (OR 1.77; 95% CI 1.43, 2.19) [13,38,49]. All three studies adjusted for age, gender, and body mass index; two studies additionally adjusted for hypertension [38,49] and the other study for diabetes mellitus [13]. Significant heterogeneity was identified for the pooled unadjusted and the age-adjusted and gender-adjusted ORs (unadjusted *I*^2^ = 69.1%, *P* = 0.002; age-adjusted and gender-adjusted *I*^2^ = 70.2%, *P* = 0.018) but not for the multivariate pooled estimate (*I*^2^ = 0.0%, *P* = 0.510). One prospective study found men with gout to have a relative risk of 2.12 (95% CI 1.22, 3.68) of developing incident nephrolithiasis over a 12-year period compared with men without gout, after adjustment for age, body mass index, diuretic use, hypertension, and dietary factors [39].

### Assessment of publication bias

When pooling the six studies describing the prevalences of CKD stage ≥3, Egger’s test yielded a borderline result of *P* = 0.05. No evidence of small study effects was seen for nephrolithiasis (Egger’s test *P* = 0.548).

## Discussion

In this systematic review and meta-analysis of population-based epidemiological studies, renal disease and nephrolithiasis were common findings in people with gout. The pooled prevalence estimates of CKD stage ≥3 and self-reported nephrolithiasis in people with gout were 24% and 14% respectively. Furthermore, after adjustment for confounding variables, people with gout were more than twice as likely to have CKD stage ≥3 (OR 2.41) and over one and a half times (OR 1.77) as likely to have ever had nephrolithiasis as people who did not have gout. Most studies employed a cross-sectional design and there was a paucity of prospective studies. The pooled unadjusted HR for incident ESRD in people with gout was 2.69; however, the lower limit of the 95% CI bordered unity. Only one study provided an adjusted HR which suggested that gout is an independent risk factor for incident ESRD (HR 1.57). Similarly, the single prospective study of the risk of developing nephrolithiasis found people with gout to have twice the risk (adjusted relative risk 2.12) of people without gout.

To our knowledge, this is the first meta-analysis of associations between gout and CKD/nephrolithiasis. Strengths of our methodology include the comprehensive search strategy and literature review process undertaken by two independent reviewers, translation of non-English-language publications, contacting study authors to request additional data to maximise the number of included studies, and an outcome which required biochemically defined CKD rather than more heterogeneous and less precise clinical diagnostic labels.

As with any systematic review, our findings are dependent upon the size and quality of the published literature. There was only a small number of studies that had examined either CKD or nephrolithiasis. Studies of CKD were all published from 2007 onwards, which is not surprising as the National Kidney Foundation CKD classification was only introduced in 2002 [19]. Widening the outcome definition to include clinical diagnostic labels for kidney disease would have increased the number of available studies but would have increased heterogeneity. Furthermore, CKD classification is widely used and highly relevant to clinical practice. The small number of published studies also raises the possibility of publication bias. Although not evident for nephrolithiasis, there were too few studies of CKD to examine for publication bias. There were several methodological limitations of the included studies worthy of further discussion. The majority of included studies were not designed with the primary aim of assessing the association between gout and CKD/nephrolithiasis. Most studies based the diagnosis of gout on physician diagnosis (which has been shown to be reasonably accurate [49]) or patient self-report rather than more rigorous methods such as the American Rheumatism Association criteria [50] or crystal identification. This limitation risks misclassification bias but, as highlighted by this study, the current evidence base offers limited numbers of epidemiological studies using more robust methods. Several studies did not include control groups without gout and only reported the prevalence of CKD/nephrolithiasis in gout but not the strength of association between the two. Although our findings demonstrate a clear independent association between gout and both CKD and nephrolithiasis, there were few prospective studies so we could not draw firm conclusions about temporal aspects of these associations. Previous epidemiological studies have shown that chronic renal disease is an independent risk factor for gout [10,11], yet there are several plausible mechanisms by which gout might predispose to CKD. Renal damage can result from co-morbid hypertension and diabetes, hyperuricaemia-mediated endothelial dysfunction and renovascular disease [55], and use of nonsteroidal anti-inflammatory drugs. Although allopurinol is widely believed to have a deleterious effect on renal function based on early observations [56], a recent systematic review suggested that it may protect against progression of CKD [57]. Inflammation in gout is increasingly recognised to persist in the inter-critical period between acute attacks [58,59], raising the possibility that inflammatory mechanisms contribute to vascular risk, as has been proposed for other inflammatory arthropathies [60]. Statistical heterogeneity was demonstrated in the meta-analyses for CKD and nephrolithiasis prevalence, the unadjusted HR between gout and incident ESRD, and the unadjusted and age-adjusted and gender-adjusted ORs between gout and nephrolithiasis. Possible sources include differences between the demographic and co-morbid characteristics of the populations studied and the different follow-up periods in the prospective studies. It is noteworthy, however, that the pooled multivariate estimates adjusting for such demographic and co-morbid factors did not demonstrate significant heterogeneity.

## Conclusions

The main clinical implications of our findings are that patients with gout should be screened for CKD and that clinicians should be made aware of the associations between gout and CKD/nephrolithiasis. Unless sought for, CKD usually progresses subclinically until reaching more advanced stages. In view of this, the significant morbidity and mortality associated with CKD [8] and the risk of CKD associated with gout, a presentation with gout in primary care should be viewed as a red flag for CKD and should prompt screening for and treatment of both CKD and its associated risk factors such as hypertension and diabetes mellitus, which are also risk factors for gout [10]. American College of Rheumatology guidelines advise considering CKD and nephrolithiasis as part of the management of gout [61] but current national and international guidelines regarding CKD [62] and nephrolithiasis [63] do not recognise gout as a risk factor for these conditions. Only one in five people presenting to primary care with acute gout are screened for CKD within a month of presentation [64].

In summary, gout is associated with both CKD and nephrolithiasis. However, there were insufficient prospective studies to determine the temporal nature of these associations or to determine the mechanisms underlying them. Whilst the associations seen suggest that clinicians should be made aware of them and that patients with gout should be screened for CKD, further studies are required to investigate these relationships further.

## Abbreviations

CI: confidence interval
CKD: chronic kidney disease
ESRD: end-stage renal disease
HR: hazard ratio
OR: odds ratio
RRT: renal replacement therapy
